# A voltage-dependent fluorescent indicator for optogenetic applications, archaerhodopsin-3: Structure and optical properties from *in silico* modeling

**DOI:** 10.12688/f1000research.10541.3

**Published:** 2017-11-15

**Authors:** Dmitrii M. Nikolaev, Anton Emelyanov, Vitaly M. Boitsov, Maxim S Panov, Mikhail N. Ryazantsev

**Affiliations:** 1 Saint-Petersburg National Research Academic University of the Russian Academy of Science, St. Petersburg, Russian Federation; 2 Saint-Petersburg State University, St. Petersburg, Russian Federation; 3 Saint-Petersburg Scientific Center of the Russian Academy of Sciences, St. Petersburg, Russian Federation

**Keywords:** optogenetics, archaerhodopsin, protein structure prediction, QM/MM, spectral tuning in rhodopsins

## Abstract

It was demonstrated in recent studies that some rhodopsins can be used in optogenetics as fluorescent indicators of membrane voltage. One of the promising candidates for these applications is archaerhodopsin-3. While it has already shown encouraging results, there is still a large room for improvement. One of possible directions is increasing the intensity of the protein's fluorescent signal. Rational design of mutants with an improved signal is an important task, which requires both experimental and theoretical studies. Herein, we used a homology-based computational approach to predict the three-dimensional structure of archaerhodopsin-3, and a Quantum Mechanics/Molecular Mechanics (QM/MM) hybrid approach with high-level multireference ab initio methodology (SORCI+Q/AMBER) to model optical properties of this protein. We demonstrated that this methodology allows for reliable prediction of structure and spectral properties of archaerhodopsin-3. The results of this study can be utilized for computational molecular design of efficient fluorescent indicators of membrane voltage for modern optogenetics on the basis of archaerhodopsin-3.

## Introduction

Precise and quick control of physiological processes using integrated optical and genetic methods is a vast area with a high number of important applications ^1^. One possible approach in this field is using fluorescent voltage-dependent indicators for detecting the activity of mammalian neurons, which allows achievement of the precision of a single neuron without perceptible time delays. It was recently shown that some rhodopsins, especially achaerhodopsin-3, can be potential candidates for such a task ^2– 5^. While the results obtained for archaerhodopsin-3 are already very encouraging, increasing the fluorescent signal of such proteins can lead to a significant progress in this field. It was also shown that the insertion of mutations into these proteins can dramatically improve the signal quality ^2– 5^.

Unfortunately, the fundamental mechanisms underlying the processes that determine fluorescence are not well understood. This lack of knowledge leads to difficulties with design of desired rhodopsin mutants. While rational design is not based on a solid foundation and, for this reason, is not very effective, another experimental approach, random mutagenesis, is very time consuming. Computational studies can provide additional insights into the problem. One of the main obstacles for computational modeling of proteins is the absence of three-dimensional structures of high quality, especially for membrane proteins, which are a challenge for crystallization. On the other hand, computational prediction of three-dimensional structures is not trivial. The goal of this study was to obtain a good-quality structure for achaerhodopsin-3, one of the most used voltage-dependent fluorescent sensors ^4, 5^, and based on this structure to predict the optical properties of this protein.

To achieve this goal, we used a homology-based computational approach for structure prediction. As the choice of a structure prediction algorithm is not straightforward, we tested several methods. To evaluate the quality of obtained structures we performed subsequent *Quantum Mechanics/Molecular Mechanics (*QM/MM) calculations of absorption maxima and compared the results with available experimental data.

## Methods

The structure of archaerhodopsin-3 was built using a homology modeling approach. Primary structures for all rhodopsins with crystallographic data are available in the Protein Data Bank library ^6^ (24 structures as of September 2016) were compared with the primary structure of archaerhodopsin-3. Archaerhodopsin-1, which has the highest sequence identity to the target protein, was chosen as a template (RCSB code 1UAZ, sequence identity 84.5%, sequence similarity 91.5%). Three algorithms of homology-based model building were tested: Medeller ^7^, I-TASSER ^8^ and RosettaCM ^9^. All methods of homology modeling heavily rely on externally made target-template alignment of primary sequences, which serves as the main instruction for model building. For this reason, we tested three algorithms of pairwise alignment using their results as an input for each method of model building. Two of the alignment methods are specifically constructed for membrane proteins, MP-T ^10^ and AlignMe ^11^, and the third one, MUSTER ^12^, gains its quality from evolutionary predictions. The latter algorithm is a built-in algorithm of I-TASSER suite and its results were used only for this method of structure prediction.

Before QM/MM calculations, several preparation steps were performed: hydrogen atoms were added using pdb2pqr package ^13^ version 2.1.1 using CHARMM force field version 27 ^14^, pH=7; hydrogen atoms were equilibrated by energy minimization in NAMD package ^15^, version 2.11. The retinal chromophore was bound to the lysine residue Lys226, whole lysine + retinal system was parameterized in CHARMM force field ^16^. The protein was inserted in the POPC membrane ^17^, the whole system was inserted in a water solvent box, the TIP3P water model ^18^ was used, the size of water box was selected so that there were at least 10 Å from any atom of protein to the edge of the system, and the system was neutralized by addition of Na ^+^ and Cl ^-^ ions.

Relaxation of the system was performed in several steps: relaxation of retinal + lysine complex with all other atoms fixed, relaxation of all atoms that were within 6 Å of the chromophore system, relaxation of whole protein and water box. During all these steps the following parameters were used: a 10 Å cutoff with switching starting at 8.5 Å was applied to the electrostatics and van der Waals interactions; Particle Mesh Ewald method ^19^ was used for dealing with electrostatics interactions, grid spacing 1Å. Equilibrated protein structure was extracted; internal waters were added into protein cavities using WaterDock program ^20^.

To calculate absorption maxima, we used the methodology that has been proven as efficient in a number of our previous studies for different kind of rhodopsins and rhodopsin mimics ^21– 27^. The structures of the archaerhodopsin-3 obtained at the previous step were optimized using two-layer ONIOM (QM:MM-EE) scheme. (QM=B3LYP/6-31G*; MM= AMBER for aminoacids and TIP3P for water, EE=electronic embedding) was implemented in the Gaussian09 package ^28^. To calculate the spectral properties of the chromophore in the presence of the protein environment (described as AMBER point charges) SORCI+Q/6-31G* level of the theory was used, as it is implemented in ORCA6.0 package ^29^. For details of previously performed QM/MM methodology see Altun *et al.* ^30, 31^.

## Results

**Figure 1.**
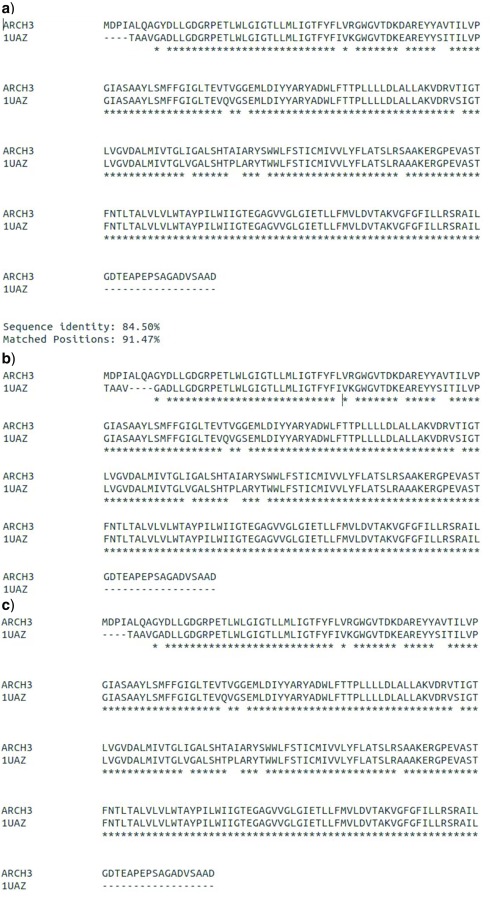
The results of the pairwise sequence alignment algorithms: **a**) AlignMe; **b**) MP-T; **c**) MUSTER.

On the first step of structure prediction of archaerhodopsin-3 we performed the alignment of its amino acid sequence with the sequence of archaerhodopsin-1, comparing three different algorithms: AlignMe, MUSTER and MP-T. The results of AlignMe and MUSTER algorithms were identical; they differ from the MP-T alignment result in the flexible N-terminal part ( Figure 1, a–c).

**Figure 2.**
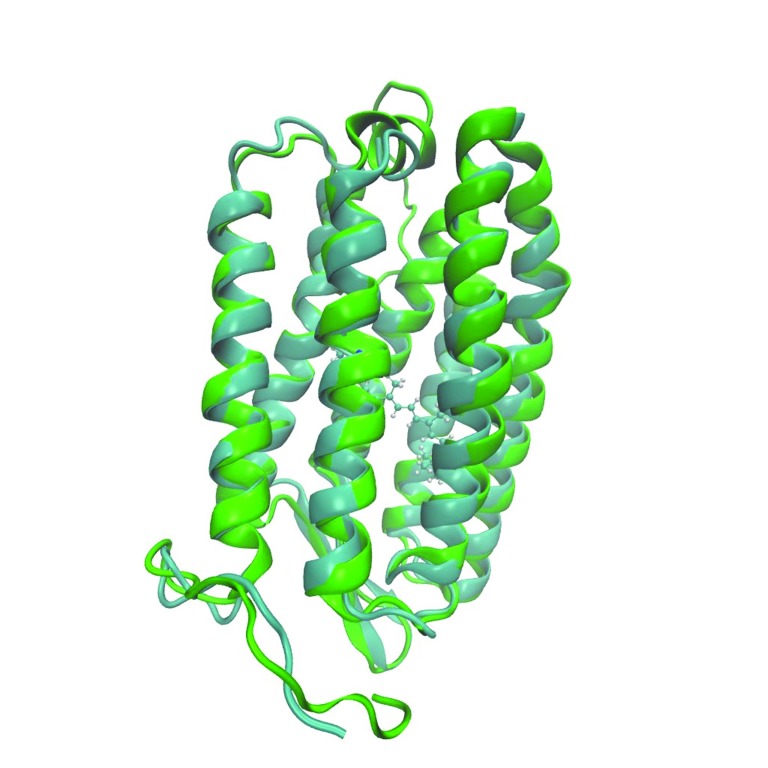
The predicted structure of archaerhodopsin-3 (green) superimposed on crystallographic structure of archaerhodopsin-1 (blue).

**Figure 3.**
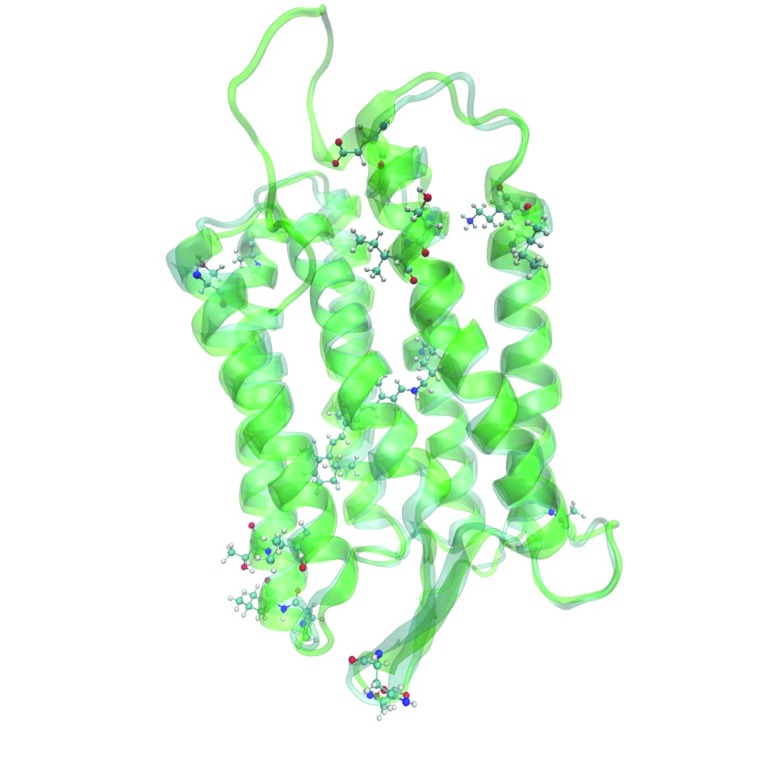
The illustration of the differences between structures of archaerhodopsin-1 and archaerhodopsin-3.

**Figure 4.**
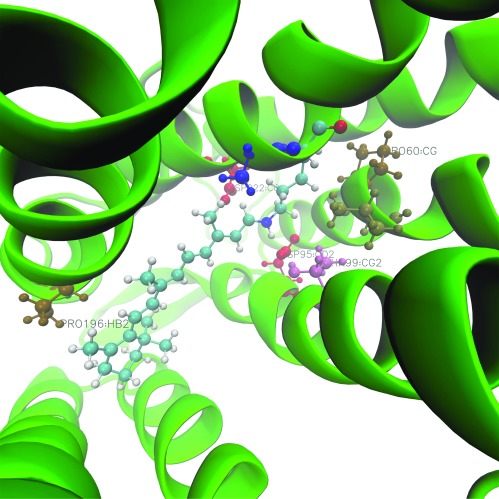
The visualization of residues, which were replaced in the site-directed mutagenesis study of archaerhodopsin-3 ^5^.

On the next step we built 7 three-dimensional models of archaerhodopsin-3 using I-TASSER, Medeller and RosettaCM algorithms of template-based structure prediction. Comparison of crystallographic structure of archaerhodopsin-1 with the predicted structure of archaerhodopsin-3 showed that these two proteins differ only in loop regions and on the edges of the alpha-helices ( Figure 2, Figure 3). These regions are located on relatively large distance from the retinal chromophore. We also highlighted the residues, which were the sites for the site-directed mutagenesis of the archaerhodopsin-3 ^5^ ( Figure 4). The computational characterization of these mutants is an interesting problem for further investigations.

For all seven predicted models we calculated the absorption maximum wavelengths. All obtained models provide reasonable results -- the deviation range is only 31 nm from the experiment ( Table 1). This deviation range is sensible considering the approximations used in our methodologies. In our previous studies, we obtained a 29 nm shift for the absorption maximum of halorhodopsin from N.pharaonis ^23^, a 25 nm shift for the absorption maximum of archaerhodopsin-2 and a 39 nm shift for the channelrhodopsin-2. The model with the smallest deviation was predicted by I-TASSER algorithm with AlignMe alignment ( Figure 2).

**Table 1.** Absorption spectrum maximum of achaerhodopsin-3 for different models.

Alignment method	Model building method	λ _max_, nm
--	Experimental wild-type structure	556
AlignMe	I-TASSER	578
MP-T	I-TASSER	581
MUSTER	I-TASSER	581
AlignMe	RosettaCM	587
MP-T	RosettaCM	585
AlignMe	Medeller	580
MP-T	Medeller	586

## Conclusions

In this study, we predicted the structure of fluorescent voltage-dependent sensor achaerhodopsin-3 and evaluated its quality with subsequent QM/MM high level *ab initio* calculations of spectral properties. The calculated absorption maximum is within 31 nm from the experimental value. Several methods of model building were tested and spectral characteristics were calculated for all resulting models. We showed that our methodology allowed for reliable prediction of optical properties of archaerhodopsin-3. The results of this study can be utilized for high-level QM/MM investigation of different aspects of photochemistry of this voltage-dependent fluorescent sensor and, therefore, to contribute in development of the efficient molecular tools for modern optogenetics.

## Data availability

The data referenced by this article are under copyright with the following copyright statement: Copyright: © 2017 Nikolaev DM et al.

The sequence of archaerhodopsin-3 was taken from Uniprot database: http://www.uniprot.org/uniprot/P96787

The template for homology modeling was taken from PDB database (rcsb code 1UAZ): http://www.rcsb.org/pdb/explore/explore.do?structureId=1UAZ

The input files of I-TASSER suite, RosettaCM, Medeller algorithms with corresponding README files, zipped output of I-TASSER suite, scripts for processing structure after homology modeling stage (with instructions in README file), input files for spectra calculations are available: doi, https://doi.org/10.5281/zenodo.830025 ^32^.

## References

[1] Deisseroth K: Optogenetics. *Nat Methods.* 2011;8(1):26–29. 10.1038/nmeth.f.324 21191368 PMC6814250

[2] Engqvist MK Mclsaac RS Dollinger P: Directed evolution of *Gloeobacter violaceus* rhodopsin spectral properties. *J Mol Biol.* 2015;427(1):205–220. 10.1016/j.jmb.2014.06.015 24979679

[3] Kralj JM Hochbaum DR Douglass AD: Electrical spiking in *Escherichia coli* probed with a fluorescent voltage-indicating protein. *Science.* 2011;333(6040):345–348. 10.1126/science.1204763 21764748

[4] Kralj JM Douglass AD Hochbaum DR: Optical recording of action potentials in mammalian neurons using a microbial rhodopsin. *Nat Methods.* 2012;9(1):90–95. 10.1038/nmeth.1782 22120467 PMC3248630

[5] Mclsaac RS Engqvist MK Wannier T: Directed evolution of a far-red fluorescent rhodopsin. *Proc Natl Acad Sci U S A.* 2014;111(36):13034–13039. 10.1073/pnas.1413987111 25157169 PMC4246972

[6] Westbrook J Feng Z Chen L: The Protein Data Bank and structural genomics. *Nucleic Acids Res.* 2003;31(1):489–491. 10.1093/nar/gkg068 12520059 PMC165515

[7] Kelm S Shi J Deane CM: MEDELLER: homology-based coordinate generation for membrane proteins. *Bioinformatics.* 2010;26(22):2833–2840. 10.1093/bioinformatics/btq554 20926421 PMC2971581

[8] Yang J Yan R Roy A: The I-TASSER Suite: protein structure and function prediction. *Nat Methods.* 2015;12(1):7–8. 10.1038/nmeth.3213 25549265 PMC4428668

[9] Song Y DiMaio F Wang RY: High-resolution comparative modeling with RosettaCM. *Structure.* 2013;21(10):1735–1742. 10.1016/j.str.2013.08.005 24035711 PMC3811137

[10] Hill JR Deane CM: MP-T: improving membrane protein alignment for structure prediction. *Bioinformatics.* 2013;29(1):54–61. 10.1093/bioinformatics/bts640 23110969

[11] Stamm M Staritzbichler R Khafizov K: AlignMe--a membrane protein sequence alignment web server. *Nucleic Acids Res.* 2014;42(Web Server issue):W246–W251. 10.1093/nar/gku291 24753425 PMC4086118

[12] Wu S Zhang Y: MUSTER: Improving protein sequence profile-profile alignments by using multiple sources of structure information. *Proteins.* 2008;72(2):547–556. 10.1002/prot.21945 18247410 PMC2666101

[13] Dolinsky TJ Nielsen JE McCammon JA: PDB2PQR: an automated pipeline for the setup of Poisson-Boltzmann electrostatics calculations. *Nucleic Acids Res.* 2004;32(Web Server issue):W665–W667. 10.1093/nar/gkh381 15215472 PMC441519

[14] Best RB Zhu X Shim J: Optimization of the additive CHARMM all-atom protein force field targeting improved sampling of the backbone φ, ψ and side-chain χ _1_ and χ _2_ dihedral angles. *J Chem Theory Comput.* 2012;8(9):3257–3273. 10.1021/ct300400x 23341755 PMC3549273

[15] Phillips JC Braun R Wang W: Scalable molecular dynamics with NAMD. *J Comput Chem.* 2005;26(16):1781–1802. 10.1002/jcc.20289 16222654 PMC2486339

[16] Zhu S Brown MF Feller SE: Retinal conformation governs p *K* _a_ of protonated Schiff base in rhodopsin activation. *J Am Chem Soc.* 2013;135(25):9391–9398. 10.1021/ja4002986 23701524 PMC5176254

[17] Klauda JF Venable RM Freites JA: Update of the CHARMM all-atom additive force field for lipids: validation on six lipid types. *J Phys Chem B.* 2010;114(23):7830–7843. 10.1021/jp101759q 20496934 PMC2922408

[18] MacKerrel AD Bashford D Bellot M: All-atom emperical potential for molecular modeling and dynamics studies of proteins. *J Phys Chem B.* 1998;102(18):3586–3616. 10.1021/jp973084f 24889800

[19] Darden TA York D Pedersen LG: Particle mesh Ewald: an *N*-log( *N*) method for Ewald sums in large systems. *J Chem Phys.* 1993;98(12):10089–10092. 10.1063/1.464397

[20] Ross GA Morris GM Biggin PC: Rapid and accurate prediction and scoring of water molecules in protein binding sites. *PLoS One.* 2012;7(3):e32036. 10.1371/journal.pone.0032036 22396746 PMC3291545

[21] Altun A Morokuma K Yokoyama S: H-bond network around retinal regulates the evolution of ultraviolet and violet vision. *ACS Chem Biol.* 2011;6(8):775–780. 10.1021/cb200100f 21650174 PMC3158842

[22] Melloni A Rossi Paccani R Donati D: Modeling, preparation, and characterization of a dipole moment switch driven by *Z/E* photoisomerization. *J Am Chem Soc.* 2010;132(27):9310–9319. 10.1021/ja906733q 20568762

[23] Ryazantsev MN Altun A Morokuma K: Color tuning in rhodopsins: the origin of the spectral shift between the chloride-bound and anion-free forms of halorhodopsin. *J Am Chem Soc.* 2012;134(12):5520–5523. 10.1021/ja3009117 22397521 PMC3786335

[24] Sinicropi A Martin E Ryazantsev M: An artificial molecular switch that mimics the visual pigment and completes its photocycle in picoseconds. *Proc Natl Acad Sci U S A.* 2008;105(46):17642–17647. 10.1073/pnas.0802376105 19004797 PMC2584735

[25] Shapiro I Ryazantsev MN Ding WJ: Computational photobiology and beyond. *Aust J Chem.* 2010;63(3):413–429. 10.1071/CH09563

[26] Schapiro I Ryazantsev MN Frutos LM: The ultrafast photoisomerizations of rhodopsin and bathorhodopsin are modulated by bond length alternation and HOOP driven electronic effects. *J Am Chem Soc.* 2011;133(10):3354–3364. 10.1021/ja1056196 21341699

[27] Sumita M Ryazantsev MN Saito K: Acceleration of the *Z* to *E* photoisomerization of penta-2,4-dieniminium by hydrogen out-of-plane motion: theoretical study on a model system of retinal protonated Schiff base. *Phys Chem Chem Phys.* 2009;11(30):6406–6414. 10.1039/b900882a 19809672

[28] Frisch MJ Trucks GW Shelegel HB: gaussian 09. Gaussian, Inc., Wallingford, CT 4,2009 Reference Source

[29] Neese FJ: A spectroscopy oriented configuration interaction procedure. *J Chem Phys.* 2003;119(18):9428–9443. 10.1063/1.1615956

[30] Altun A Yokoyama S Morokuma K: Spectral tuning in visual pigments: an ONIOM(QM:MM) study on bovine rhodopsin and its mutants. *J Phys Chem B.* 2008;112(22):6814–6827. 10.1021/jp709730b 18473437 PMC2491561

[31] Altun A Yokoyama S Morokuma K: Mechanism of spectral tuning going from retinal in vacuo to bovine rhodopsin and its mutants: multireference ab initio quantum mechanics/molecular mechanics studies. *J Phys Chem B.* 2008;112(51):16883–16890. 10.1021/jp807172h 19367945 PMC2669894

[32] Nikolaev DM Emelyanov A Boitsov VM: Supplementary information for: “A voltage-dependent fluorescent indicator for optogenetic applications, archaerhodopsin-3: Structure and optical properties from *in silico* modeling” [Data set]. *Zenodo.* 2017 Data Source 10.12688/f1000research.10541.1 PMC5381632 28435665

