# Supplementary material for: A voltage-dependent fluorescent indicator for optogenetic applications, archaerhodopsin-3: Structure and optical properties from in silico modeling
Source: F1000Res. 2017 Nov 15;6:33. Originally published 2017 Jan 11. [Version 3] doi: 10.12688/f1000research.10541.3 (PMC5381632; doi:10.12688/f1000research.10541.3)
Supplement: Peer review history

# Peer review: Referee response for version 3

**DOI:** 10.5256/f1000research.14321.r28011

**Published:** 2017-11-22

**Authors:** Jeremy N. Harvey

**Affiliations:** 1 Division of Quantum Chemistry and Physical Chemistry, Department of Chemistry, KU Leuven, Leuven, Belgium

I am satisfied by the authors' responses.

I have read this submission. I believe that I have an appropriate level of expertise to confirm that it is of an acceptable scientific standard.

# Peer review: Referee response for version 3

**DOI:** 10.5256/f1000research.14321.r28010

**Published:** 2017-11-22

**Authors:** Adam E. Cohen

**Affiliations:** 1 Department of Chemistry and Chemical Biology, Harvard University, Cambridge, MA, USA

The authors have improved the manuscript in response to the comments from the other referees and me.

I have read this submission. I believe that I have an appropriate level of expertise to confirm that it is of an acceptable scientific standard.

# Peer review: Referee response for version 2

**DOI:** 10.5256/f1000research.11514.r20540

**Published:** 2017-03-31

**Authors:** Jeremy N. Harvey

**Affiliations:** 1 Division of Quantum Chemistry and Physical Chemistry, Department of Chemistry, KU Leuven, Leuven, Belgium

The very short manuscript reports homology modelling of the membrane protein, coupled with 'validation' by using vertical excitation energies computed with a QM/MM protocol. I think the work is interesting, particularly since the authors have made their data available, making it easier to others to build upon it. However, I have a number of concerns, many of which could be addressed simply by describing the results and their implications in a bit more detail.

1. The first set of comments (1)-(3) in Cohen & Farhi's report seem important to me. I think that in the paper, the authors use the position of the absorption peak to judge the validity of their structural model. So the precise mechanism of fluorescence does not matter, nor does proton transfer, nor does the fluorescence quantum yield. Rather these are ideas for future work. But it would do no harm to spell these things out a bit more.
2. There is *very* little in the results section: 1 Figure, 1 Table, and 4 lines of text. Why not tell us a bit more? For example, the authors generate a homology model, but apart from showing us a cartoon structural picture of it, they don't say anything about it. How good was the sequence alignment? How long are the various helices? How well does this compare to various other transmembrane proteins of this type? How much did the other homology models differ from the preferred one, and from each other? Without this kind of analysis, this paper really just consists of a set of input and output files of homology modelling/sequence alignment.
3. Still in the results section, the authors use SORCI+Q QM/MM vertical excitation energies computed at optimized structures to assess the quality of the homology models. This seems extremely naive to me. The different models return vertical excitation energies that differ by just over 1%, and are about 4% from experiment. I really don't think that this is a good basis for deciding that one of them is better than the others. There are just too many sources of error for making this a meaningful comparison. On the one hand, SORCI+Q with the basis set used will potentially have an error of several tenths of an eV. 0.2 eV is 10% of the excitation energy based on the experimental spectrum. Furthermore, the authors have computed just one vertical excitation energy for each model, at a structure derived by a rather minimal geometry optimization ('relaxation') protocol. The FWHM of the UV/Vis absorption peak of bacetriorhodopsin appears to be about 100nm, i.e. 15% of the band centre. I assume roughly the same is true here. In principle, even accepting that the starting point homology model is correct, and the SORCI Hamiltonian is exact, the QM/MM protocol used could return a vertical excitation energy 50 nm or so either side of the experimental peak. This just makes the criterion meaningless. There are other criteria for assessing the internal consistency of homology models.

As I understand it, the philosophy of F1000Research is that papers can be published if they have some interest, and I think some people might find the homology modelling interesting, and the detailed structural models with the chromophore. So I'm happy for this to be indexed. But I think the QM/MM calculations are hugely over-interpreted, and recommend the authors consider revising this section, as well as adding some of the discussion I mention above.

I have read this submission. I believe that I have an appropriate level of expertise to confirm that it is of an acceptable scientific standard, however I have significant reservations, as outlined above.

# Response

**Published:** 2017-11-10

**Authors:** Mikhail N. Ryazantsev

**Affiliations:** Saint-Petersburg State University, Russia, Russian Federation

Dear Prof. Harvey,

Thank you for your review of our article “A voltage-dependent fluorescent indicator for optogenetic applications, archaerhodopsin-3: Structure and optical properties from *in silico* modeling”. We have carefully read it and made several changes of the article, considering your notices. We attach the new version of the article in this email.

1. “ There is *very* little in the results section: 1 Figure, 1 Table, and 4 lines of text. Why not tell us a bit more? For example, the authors generate a homology model, but apart from showing us a cartoon structural picture of it, they don't say anything about it. How good was the sequence alignment? How long are the various helices? How well does this compare to various other transmembrane proteins of this type? How much did the other homology models differ from the preferred one, and from each other? Without this kind of analysis, this paper really just consists of a set of input and output files of homology modelling/sequence alignment.”

Considering this remark, we edited the results section of our article. The sequence identity between Arch-1 and Arch-3 is 84.5%, sequence similarity is 91.5% (based on the AlignMe alignment of these sequences). We added this information in the “Methods” section of our article.

We also compared the results of the sequence alignment algorithms. The comparison showed that all three of them are very similar: AlignMe and MUSTER gave identical results with small difference from the MP-T alignment in the beginning of the sequence (Figure 1 in the article). We added the following text in the “Results” section of our article:

 “On the first step of structure prediction of archaerhodopsin-3 we performed the alignment of its amino acid sequence with the sequence of archaerhodopsin-1, comparing three different algorithms: AlignMe, MUSTER and MP-T. The results of AlignMe and MUSTER algorithms were identical; they differ from the MP-T alignment result in the flexible N-terminal part (Figure 1, a-c).”

We also compared the crystallographic structure of archaerhodopsin-1 and the predicted model of archaerhodopsin-3 (Figure 2, 3 in the article). We showed that the structures of two proteins are very similar, and they differ only in loop regions and on the edges of alpha-helices, i.e. in the regions located quite far from the chromophore. We added the following text in the “Results” section of our article:

“Comparison of crystallographic structure of archaerhodopsin-1 with the predicted structure of archaerhodopsin-3 showed that these two proteins differ only in loop regions and on the edges of the alpha-helices (Figure 2, 3). These regions are located on relatively large distance from the retinal chromophore.”

2. *“Still in the results section, the authors use SORCI+Q QM/MM vertical excitation energies computed at optimized structures to assess the quality of the homology models. This seems extremely naive to me. The different models return vertical excitation energies that differ by just over 1%, and are about 4% from experiment. I really don't think that this is a good basis for deciding that one of them is better than the others. There are just too many sources of error for making this a meaningful comparison. On the one hand, SORCI+Q with the basis set used will potentially have an error of several tenths of an eV. 0.2 eV is 10% of the excitation energy based on the experimental spectrum. Furthermore, the authors have computed just one vertical excitation energy for each model, at a structure derived by a rather minimal geometry optimization ('relaxation') protocol. The FWHM of the UV/Vis absorption peak of bacetriorhodopsin appears to be about 100nm, i.e. 15% of the band centre. I assume roughly the same is true here. In principle, even accepting that the starting point homology model is correct, and the SORCI Hamiltonian is exact, the QM/MM protocol used could return a vertical excitation energy 50 nm or so either side of the experimental peak. This just makes the criterion meaningless. There are other criteria for assessing the internal consistency of homology models.”*

To refer to these comments, we modified the text. In this case, all models provide reasonable results. However, it is not always the point, and predicted structures can give easily more than 50 nm off experimental values after a relatively small change of three-dimensional structure. In other words, modeling of absorption maxima is very sensitive to the quality of three-dimensional structures.

Considering these remarks, we added the following modifications in the “Results” section:

“All obtained models provide reasonable results – the deviation range is only 31 nm from the experiment (Table 1). This deviation range is sensible considering the approximations used in our methodologies. In our previous studies, we obtained a 29 nm shift for the absorption maximum of halorhodopsin from N.pharaonis, a 25 nm shift for the absorption maximum of archaerhodopsin-2 and a 39 nm shift for the channelrhodopsin-2.”

# Peer review: Referee response for version 2

**DOI:** 10.5256/f1000research.11514.r20866

**Published:** 2017-03-20

**Authors:** Adam E. Cohen, Samouil Farhi

**Affiliations:** 1 Department of Chemistry and Chemical Biology, Harvard University, Cambridge, MA, USA; 2 Harvard University, Cambridge, MA, USA

Nikolaev et al. attempt to predict archaerhodopsin-3 structural and optical properties using *in silico* approaches, layering QM/MM calculations of absorption spectra over homology based structural modeling. As the authors note, such work would be invaluable for guiding the design of new archaerhodopsin variants, with potential broad applicability in the fields of optogenetics and voltage imaging.

 The creation of a ground-state homology model is a useful first step, but the authors should acknowledge and discuss some of the challenges that would need to be addressed for this model to be useful in predicting voltage-sensitive fluorescent properties. These are:

1. The fluorescence of wild-type Arch-3 appears not to come from the ground state, but from a photogenerated intermediate (see ^1^). Thus a model would need to adopt the correct retinal isomerization state and opsin conformation to reproduce the fluorescence.
2. To predict voltage sensitivity, a model would need to incorporate the intra-membrane electric field and its influence on the motion of protons within the structure.
3. The fluorescence quantum yield depends on the branching ration in the electronic excited state between radiative and non-radiative decay pathways. A prediction of quantum yield would need to include simulation of these excited state dynamics.

There are several other ways in which the manuscript could be improved:

1. The abstract states, “the fluorescent signal for wild-type achaerhodopsin-3 is not strong enough for real applications.” The first paragraph repeats this claim. This is an overly pessimistic view. Many papers have used wild-type Archaerhodopsin 3 (note spelling) and its mutants for fluorescent voltage imaging. A more accurate statement would be, “An increase in the fluorescence of Archaerhodopsin 3 would broaden the scope of its possible applications.”
2. Arch-1 was used as a homology model for Arch-3.How similar are the two proteins? What percentage sequence identity and sequence homology?
3. The sole figure shows the best output structure, but doesn’t compare it to anything. It should at least be superimposed with the Arch-1 input structure. The outputs of the different alignment methods should also be compared and any differences highlighted. It would also be useful to have more details on where the Arch-1 and Arch-3 sequences differ - perhaps a linear sequence alignment and a figure showing where differences occur in the 3-D structure.
4. The authors reference several papers which have performed site-directed mutagenesis on this protein. They should highlight those residues on their output structure. Ideally, they should also perform their AlignMe/I-TASSER analysis on one of the well characterized mutants to show if their model has predictive value.
5. For scientists unfamiliar with the possibilities of in silico modeling, it is difficult to evaluate whether an 18 nm deviation in absorbance maximum is a lot or a little. It would be helpful to give context using other, previously studied scaffolds. How accurately can the absorbance maximum of eGFP be determined? Of another membrane protein, such as channelrhodopsin-2?
6. The authors admirably package and make available all of the inputs and outputs of their calculations. Unfortunately, the abstruse organization of the data makes it difficult to extract useful information from their data. The root directory of the supplemental data should include a single clearly written readme.txt file explaining how to access the output structures of each of their homology models. The output files should be obviously labeled - meaning “Figure1structure” not “P96787_wi” - and be in a commonly used file format, such as .pdb.

We have read this submission. We believe that we have an appropriate level of expertise to confirm that it is of an acceptable scientific standard, however we have significant reservations, as outlined above.

## References

[1] MaclaurinDVenkatachalamVLeeHCohenAE: Mechanism of voltage-sensitive fluorescence in a microbial rhodopsin. *Proc Natl Acad Sci U S A*.2013;110(15) : 10.1073/pnas.1215595110 5939-44 10.1073/pnas.1215595110 23530193PMC3625274

# Response

**Published:** 2017-11-10

**Authors:** Mikhail N. Ryazantsev

**Affiliations:** Saint-Petersburg State University, Russia, Russian Federation

Dear Prof. Cohen and Samouil Farhi.

Thank you for your review of our article “A voltage-dependent fluorescent indicator for optogenetic applications, archaerhodopsin-3: Structure and optical properties from *in silico* modeling”. We have carefully read it and made the required changes in the article.

1. *The abstract states, “the fluorescent signal for wild-type achaerhodopsin-3 is not strong enough for real applications.” The first paragraph repeats this claim. This is an overly pessimistic view. Many papers have used wild-type Archaerhodopsin 3 (note spelling) and its mutants for fluorescent voltage imaging. A more accurate statement would be, “An increase in the fluorescence of Archaerhodopsin 3 would broaden the scope of its possible applications.”*

To refer to this comment, we added the following changes into the abstract:

“One of the promising candidates for these applications is archaerhodopsin-3. While it has already shown encouraging results, there is still a large room for improvement. One of possible directions is increasing the intensity of the protein's fluorescent signal.”

In the first paragraph we made the similar changes:

“While the results obtained for archaerhodopsin-3 are already very encouraging, increasing the fluorescent signal of such proteins can lead to a significant progress in this field.”

2. *“Arch-1 was used as a homology model for Arch-3.How similar is the two proteins? What percentage sequence identity and sequence homology?”*

Arch-1 and Arch-3 are very evolutionally close. We added this information in the “Methods” section of the new version of our article: “sequence identity 84.5%, sequence similarity 91.5%”.

3. *“The sole figure shows the best output structure, but doesn’t compare it to anything. It should at least be superimposed with the Arch-1 input structure. The outputs of the different alignment methods should also be compared and any differences highlighted. It would also be useful to have more details on where the Arch-1 and Arch-3 sequences differ - perhaps a linear sequence alignment and a figure showing where differences occur in the 3-D structure.”*

To refer to this comment, we edited the “Results” section of our article. We added the comparison of outputs of the three alignment methods we used in our work. We showed that the results of AlignMe and MUSTER algorithms are identical, and they only slightly (in the beginning of the sequences) differ from the MP-T alignment results (Figure 1, a-c in the article). We added the following part into our article:

“The results of AlignMe and MUSTER algorithms were identical; they differ from the MP-T alignment result in the flexible N-terminal part (Figure 1, a-c).”

We also compared the crystallographic structure of archaerhodopsin-1 and the predicted model of archaerhodopsin-3 (Figure 2, 3 in the article). We showed that the structures of two proteins are very similar, and they differ only in loop regions and on the edges of alpha-helices, i.e. in the regions located quite far from the chromophore. We added the following text in the “Results” section of our article:

“Comparison of crystallographic structure of archaerhodopsin-1 with the predicted structure of archaerhodopsin-3 showed that these two proteins differ only in loop regions and on the edges of the alpha-helices (Figure 2, 3). These regions are located on relatively large distance from the retinal chromophore.”

4. “ *The authors reference several papers which have performed site-directed mutagenesis on this protein. They should highlight those residues on their output structure. Ideally, they should also perform their AlignMe/I-TASSER analysis on one of the well characterized mutants to show if their model has predictive value.”*

We highlighted the residues, which were the sites for the site-directed mutagenesis of the archaerhodopsin-3 (Mclsaac RS et al,  PNAS, 2014) (Figure 4 in the article). The computational characterization of mutants is an interesting problem and we will consider it in our research. We hope to publish an article about computational investigation of different mutants of both archaerhodopsin-3 and Gloebacter violaceus rhodopsin.

We added the following text in the “Results” section of our article:

“We also highlighted the residues, which were the sites for the site-directed mutagenesis of the archaerhodopsin-3 ^5^ (Figure 4). The computational characterization of these mutants is an interesting problem for further investigations.”  

5. *“For scientists unfamiliar with the possibilities of in silico modeling, it is difficult to evaluate whether an 18 nm deviation in absorbance maximum is a lot or a little. It would be helpful to give context using other, previously studied scaffolds. How accurately can the absorbance maximum of eGFP be determined? Of another membrane protein, such as channelrhodopsin-2?”*

In this study, all models provide reasonable results for the values of absorption maxima. Considering the approximations we use in the QM/MM methodologies, the deviation range up to 40 nm is considered to be good; however, in complicated cases we can obtain a 50-60 nm shift from the experimental value. Modeling of the absorption maxima is very sensitive to the quality of three-dimensional structures, and even relatively small changes of the structures can lead to huge shifts of this value.

In the case of the microbial rhodopsins we obtained many results for the absorption maxima of proteins with available crystallographic structure. For example, we obtained a 29 nm shift for halorhodopsin from N. pharaonis, a 25 nm shift for archaerhodopsin-2 and a 39 nm shift for the channelrhodopsin-2. These results lie in the same deviation range, and for this reason we think that the models we obtained have quite high quality. Substantial errors in the models would lead to much bigger shifts (up to 100-200 nm).

Considering these remarks, we added the following modifications in the “Results” section:

“This deviation range is sensible considering the approximations used in our methodologies. In our previous studies, we obtained a 29 nm shift for the absorption maximum of halorhodopsin from N.pharaonis ^23^, a 25 nm shift for the absorption maximum of archaerhodopsin-2 and a 39 nm shift for the channelrhodopsin-2. The model with the smallest deviation was predicted by I-TASSER algorithm with AlignMe alignment (Figure 2).”

5 *. “The authors admirably package and make available all of the inputs and outputs of their calculations. Unfortunately, the abstruse organization of the data makes it difficult to extract useful information from their data. The root directory of the supplemental data should include a single clearly written readme.txt file explaining how to access the output structures of each of their homology models. The output files should be obviously labeled - meaning “Figure1structure” not “P96787_wi” - and be in a commonly used file format, such as .pdb.”*

Considering this remark, we created a new version of the archive:

https://zenodo.org/record/830025#.Wcg29Fd2O1E

# Peer review: Referee response for version 2

**DOI:** 10.5256/f1000research.11514.r19223

**Published:** 2017-01-31

**Authors:** Kirill A. Afonin

**Affiliations:** 1 Nanoscale Science Program, Department of Chemistry, The Center for Biomedical Engineering and Science (CBES), The University of North Carolina at Charlotte, Charlotte, NC, USA

Overall the paper by Nikolaev et al., is well written and an interesting part of the photo- and biochemical realm. The paper provides very promising results and a unique way of analyzing structure functionality. I am interested to see what other results arise and the how the applications indicated can be strengthened. I have just a couple of minor suggestions that may help to understand the described subject a bit better.

- In your abstract please name some examples of real applications.
- The methodology and computational analysis was a little over my head and difficult to follow. However, the proper audience for this paper may be for those with a stronger foundation of computational analysis and programs indicated in the paper.
- Archaerhodopsin-1 is misspelled on page 2.

Best wishes to this group and any further work that will be done!

I have read this submission. I believe that I have an appropriate level of expertise to confirm that it is of an acceptable scientific standard.
